# CMV and Triggers of Systemic Sclerosis

**DOI:** 10.1371/journal.pmed.0030045

**Published:** 2005-12-06

Systemic sclerosis (SSc) is an autoimmune disease characterized by structural and functional vascular abnormalities, immunologic changes, and excessive extracellular matrix deposition, leading to fibrosis of the skin and the internal organs. The activation of the immune system is vital in the pathogenesis of SSc. Autoantibodies directed against cell surface antigens are believed to induce endothelial cell damage and apoptosis, which are thought to be a primary event in the pathogenesis of the disease, leading to a cascade of stimulatory changes involving many cells, including fibroblasts, T lymphocytes, and macrophages. In turn, these activated cells secrete substances, including cytokines and their soluble receptors, and enzymes and their inhibitors. These substances lead to changes in the extracellular matrix compounds, including fibronectin, proteoglycans, and collagen types I, III, V, and VII; specifically TGF-beta, interleukin-4 (IL-4), and platelet-derived growth factor are profibrotic cytokines.

Environmental factors may be involved in the disease's pathogenesis. Previous research has shown that a molecular mimicry mechanism links antibodies against the human cytomegalovirus (hCMV)-derived protein UL94 to the pathogenesis of SSc. The UL94 epitope shows homology with NAG-2, a surface molecule highly expressed on endothelial cells. Anti-UL94 peptide antibodies have been shown to induce apoptosis of endothelial cells upon engagement with the NAG-2-integrin complex. [Fig pmed-0030045-g001]

**Figure pmed-0030045-g001:**
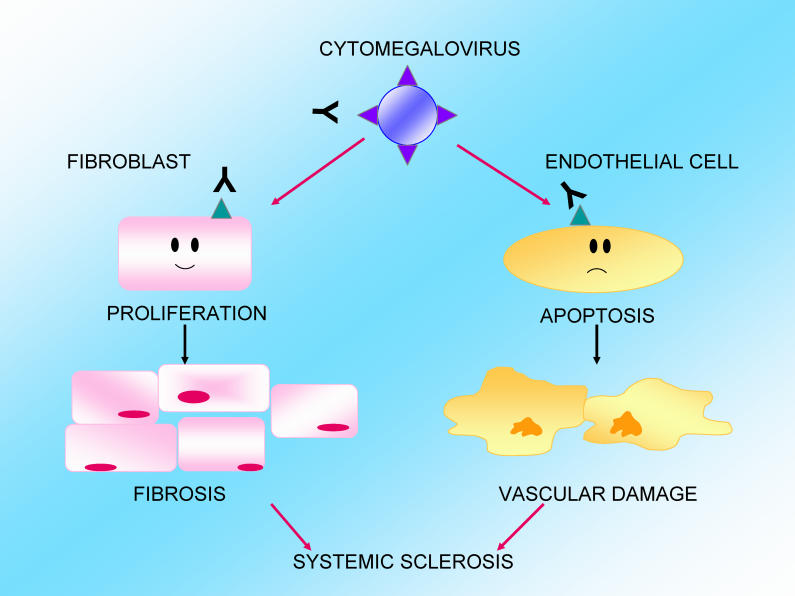
Cytomegalovirus in the pathogenesis of systemic sclerosis

In a new study, Claudio Lunardi and colleagues examined further whether hCMV could be involved in the pathogenesis of fibrosis, and also attempted to dissect out the molecular mechanisms behind the disease. They found that NAG-2 was also expressed on dermal fibroblasts, and that anti-UL94 antibodies bind to fibroblasts.

Using gene arrays, they analyzed the transcriptional profile in endothelial cells and dermal fibroblasts in response to treatment with antibodies against the UL94 peptide. Exposure of endothelial cells to anti-UL94 antibodies had a profound impact on gene expression, resulting in the upregulation of 1,645 transcripts. The genes altered encoded for adhesion molecules, chemokines, colony-stimulating factors, growth factors, and molecules involved in apoptosis. Dermal fibroblasts showed an upregulation of 989 transcripts and acquired a “scleroderma-like” phenotype, with upregulation of genes involved in extracellular matrix deposition, growth factors, chemokines, and cytokines.

To confirm these findings, the investigators measured the levels of chemokines, cytokines, growth factors, and collagen type I in the supernatants of stimulated and unstimulated cells. They found that the concentration of the molecules was higher in the cells incubated with anti-hCMV antibodies, confirming that genes' upregulation was paralleled by the induction of protein synthesis.

Taking this analysis further into patients, the investigators measured the serum concentrations of some cytokines, chemokines, and adhesion molecules in patients and controls, and confirmed that the genes found overexpressed in vitro following stimulation with anti-hCMV antibodies could indeed be of relevance in vivo.

Altogether, these findings suggest that these cross-reacting antivirus antibodies were able to induce not only endothelial cell activation and apoptosis but also fibroblast activation. They could thus be a single trigger of the two hallmarks of SSc, vascular damage and fibrosis.

